# Current Research for Heart Disease Biology and Therapeutics

**DOI:** 10.3390/ijms251910744

**Published:** 2024-10-06

**Authors:** Claudia Kusmic

**Affiliations:** Institute of Clinical Physiology, National Research Council (CNR), 56124 Pisa, Italy; claudia.kusmic@cnr.it; Tel.: +39-050-315-2669

Cardiac and vascular diseases are the leading cause of death globally. Heart disease covers a wide range of structural and functional changes that affect the heart, including conditions like coronary artery disease, abnormal heart rhythms, congenital heart defects, valve disease, and heart failure (HF). In many cases, less severe forms of heart disease have the potential to advance or intersect with other conditions, possibly leading to a more significant pathological outcome, including severe HF. Heart failure is a significant public health challenge due to its high prevalence and severe impact on patients and healthcare systems. It affects about 2% of the global population and is expected to increase due to ageing populations and improved survival rates from other cardiac conditions. By 2030, it is projected that nearly 50% of the population over 60 years of age will be affected by HF [1,2]. Cardiac research has increasingly focused on exploring new and innovative approaches to better understand the biological basis of cardiac disease onset and progression, as well as to develop novel therapeutic approaches that target molecular pathways involved in the transition to pathophysiological conditions.

This Special Issue of the *International Journal of Molecular Science* features four research articles and six reviews that cover various aspects of cardiac pathophysiology. These contributions include studies of basic research and therapeutic and functional approaches. They also provide new insights into potential molecular targets for therapeutic strategies and new diagnostic and prognostic markers in heart disease.

Two reviews specifically address different aspects of acute myocardial infarction (MI). In particular, Salvadori and colleagues thoroughly analyse and critically discuss the latest research on epigenetic changes following myocardial infarction (MI). They specifically examine the role of miRNAs and their interaction with important signalling pathways in the different early stages of the healing process after an ischemic event, namely the inflammation, resolution, and maturation phases [3]. Additionally, they shed light on the differences in the risk factors and long-term outcomes for MI between males and females.

Zheng et al. revisit the role of vitamin C, a well-established antioxidant that reduces oxidative stress [4]. The beneficial role of vitamin C in the cardiovascular system remains controversial, and previous studies on the association between vitamin C levels and MI have shown conflicting results. Although vitamin C demonstrates a strong antioxidant function at both the cellular and organ levels, further studies are needed to provide time-dependent cumulative dose data to clarify its role in the prevention and/or treatment of cardiovascular diseases. This review article offers a comprehensive perspective on the potential of vitamin C as a preventive supplement and/or as part of precise and targeted therapeutics in cardiovascular management therapy.

The review article authored by Lofrumento et al. provides an insightful analysis of the off-target effects of P2Y12 receptor inhibitors in the context of cardiac fibrosis during the remodelling process following MI [5]. P2Y12 is a platelet purinergic receptor for adenosine 5′-diphosphate (ADP), significantly contributing to arterial thrombosis. However, multiple studies have consistently shown that P2Y12 inhibitors, in addition to their antithrombotic effects, may also have extra adenosine-mediated effects as they are present in various cell types, including endothelial and immune cells. This suggests that P2Y12 receptor inhibitors may impact molecular pathways related to cardiac remodelling and heart failure. The review focuses on how adenosine may interact with and stimulate various pathways involved in cardiac fibrosis, such as the Wnt/β-catenin signalling pathway.

Moving into the field of non-ischemic cardiomyopathies, Zhang and Dhalla provide a comprehensive review of the impact of pro-inflammatory cytokines in the pathogenesis of various manifestations of heart disease, including atherosclerosis, acute MI, cardiac remodelling, and ventricular and atrial arrhythmias [6]. Despite advancements in understanding the role of pro-inflammatory cytokines in heart diseases, it is still uncertain whether they will be targets for therapies or continue to serve as biological markers or risk predictors of disease states.

Thorkelsson and Chin discuss the role of alpha-B-crystallin protein in cardiomyopathic disease in their review article [7]. Alpha-B-crystallin is a member of the heat shock family of proteins and plays a role in maintaining cardiac homeostasis. However, mutations in this protein are associated with heart diseases such as hypertrophic cardiomyopathy, restrictive cardiomyopathy, and myofibrillar myopathy in humans. The article discusses these mutations and their impact on the structure and function of the protein. It also delves into the use of mouse models to gain information on the mechanisms and signalling pathways affected by these mutations and to identify potential critical molecular targets for therapeutic interventions.

The research article by Sunagawa et al. explores the anti-hypertrophic effect of anserine in primary cultured cardiomyocytes and a mouse model of heart failure [8]. Anserine, similar to carnosine, is a naturally occurring histidine-containing dipeptide. The findings suggest that anserine helps reduce the development of cardiac hypertrophy and systolic dysfunction by inhibiting p300-histone acetyltransferase (HAT). This enzyme is a transcriptional coactivator in the nuclei of cardiomyocytes and is associated with pathological cardiomyocyte hypertrophy and the development of cardiac dysfunction. The authors propose that anserine could be a valuable compound for preventing HF.

Concerns regarding lithium (Li) toxicity and its negative impact on the heart have inspired the study by L’Abbate and colleagues [9]. Li is commonly used to treat bipolar disorders and is also a rapidly growing environmental pollutant. The findings indicate that giving mice a dose of lithium equivalent to the therapeutic human dose for 12 weeks resulted in significant impairment in heart function, ventricular repolarisation, and increased arrhythmias under adrenergic stimulation. Additionally, Li treatment caused an increase in the size of cardiomyocytes and affected signalling pathways associated with hypertrophy. The study also identified some abnormal histopathological changes in the liver and kidneys.

Regarding the issue of cardiotoxicity, Picchio and colleagues assessed the cardiotoxic effects of radiotherapy by studying the characteristics and paracrine behaviour of resident cardiac mesenchymal stromal cells (CMSCs) in rats [10]. These cells were isolated 6 and 12 weeks after the completion of radiotherapy. The study found that CMSCs showed several abnormal features in a dose- and time-dependent manner, with the most severe outcomes seen in those exposed to the highest dose over time. The findings suggest that early changes in paracrine activity and gene expression in the cardiac stroma may support the delayed cardiac dysfunction phenotype observed in vivo.

Moving on to the field of arrhythmic heart disease, in their review article, Föster and colleagues thoroughly examine the intricate relationship between atrial fibrillation (AF) and obesity [11]. They explore the combined impact of weight loss and catheter ablation (CA) on atrial remodelling and atrial fibrillation, shedding light on the potential synergistic effects of these interventions. CA is an established AF treatment to restore sinus rhythm. Regrettably, clinical evidence highlighted that AF patients with obesity undergoing CA are more likely to experience AF recurrence. In their article, the authors also discuss the potential of specific circulating miRNAs as biomarkers and argue how they can guide CA treatment strategies in obese patients affected by AF.

Remaining in the field of atrial dysfunction, the research study by Cebro-Márquez and colleagues has identified the miRNA 486-5p as a circulating biomarker that holds significant prognostic value for patients with AF referred for ablation due to broad low-voltage area (LVA) maps [12]. The LVA extension is acknowledged as a surrogate index for assessing the degree of atrial fibrosis. In a cohort of 44 patients who were referred for AF ablation and categorised according to LVA extension, the authors found that out of the 84 miRNAs identified as being differentially expressed in the peripheral blood of patients with varying degrees of LVA, only the miRNA 486-5p showed potential as a reliable predictor of LVA percentage.

Overall, the ten articles featured in this Special Issue provide informative and representative snapshots of current issues in the field. They showcase the ongoing strong interest in the biology of heart disease and the need to explore new and innovative therapeutic approaches. I am thankful for the opportunity to present our readers with such a valuable collection of studies.

I want to express my gratitude to all the contributors to this Special Issue, as well as to the Editorial Board and the assistant editors of the *International Journal of Molecular Sciences* for their invaluable support.
